# Antiasthmatic activity of *Moringa oleifera* Lam: A clinical study

**DOI:** 10.4103/0253-7613.40486

**Published:** 2008

**Authors:** Babita Agrawal, Anita Mehta

**Affiliations:** Department of Pharmacology, L.M. College of Pharmacy, Ahmedabad - 380 009, Gujarat, India

**Keywords:** Bronchial asthma, *Moringa oleifera*, pulmonary function tests

## Abstract

The present study was carried out to investigate the efficacy and safety of seed kernels of *Moringa oleifera* in the treatment of bronchial asthma. Twenty patients of either sex with mild-to-moderate asthma were given finely powdered dried seed kernels in dose of 3 g for 3 weeks. The clinical efficacy with respect to symptoms and respiratory functions were assessed using a spirometer prior to and at the end of the treatment. Hematological parameters were not changed markedly by treatment with *M. oleifera*. However, the majority of patients showed a significant increase in hemoglobin (Hb) values and Erythrocyte sedimentation rate (ESR) was significantly reduced. Significant improvement was also observed in symptom score and severity of asthmatic attacks. Treatment with the drug for 3 weeks produced significant improvement in forced vital capacity, forced expiratory volume in one second, and peak expiratory flow rate values by 32.97 ± 6.03%, 30.05 ± 8.12%, and 32.09 ± 11.75%, respectively, in asthmatic subjects. Improvement was also observed in % predicted values. None of the patients showed any adverse effects with *M. oleifera*. The results of the present study suggest the usefulness of *M. oleifera* seed kernel in patients of bronchial asthma.

Bronchial asthma is a syndrome, characterized by increased responsiveness of trachea and bronchi to various stimuli and manifested by acute, recurrent, and chronic attacks of widespread narrowing of airways. Clinically, asthma is expressed by airway obstruction that involves inflammation of the pulmonary airways and bronchial hyperresponsiveness that is usually reversible.[1] The past decade has witnessed phenomenal increases in the incidences of asthma, asthma-related deaths, and hospitalization. Existing classes of antiasthmatic agents offer a limited variety of action that can be combined in a complementary and additive manner. Individual oral agents act only on a part of the pathogenic process of bronchial asthma. Hence, they may not produce any cure and may not prevent all complications of bronchial asthma. Ayurveda has recommended number of drugs from indigenous plant sources for the treatment of bronchial asthma and other allergic disorders. *Moringa oleifera* Lam. (Fam: *Moringaceae*) (*M. oleifera*) is one such drug, used by many ayurvedic practitioners for the treatment of asthma and chronic rheumatism.[2]

*Moringa oleifera* is a medium-sized tree, found wild in the sub-Himalayan tract. The plant is reported to elicit good clinical response in children suffering from upper respiratory tract infection and skin infection. Seeds reportedly have purgative, antipyretic,[3] antispasmodic, anti-inflammatory,[4,5] and diuretic activity.[6] It has been reported that alkaloid from the plant closely resembles ephedrine in action and useful in the treatment of asthma. Alkaloid *Moringine* relaxes bronchioles.[7] However, no clinical studies have so far been carried out with respect to its efficacy in bronchial asthma. Thus, the objective of the present investigation was to carry out clinical evaluation of *M. oleifera* to assess its therapeutic potential in asthma.

## Materials and Methods

Seed kernels of *M. oleifera* were purchased from the local market of Ahmedabad and were identified and authenticated by Department of Pharmacognosy, L.M. College of Pharmacy, Ahmedabad, India. The seeds were powdered and sachets were prepared. An open label, noncomparative clinical study was carried out on 20 patients (17-70 years) of either sex, having mild-to-moderate bronchial asthma,[8] visiting Outpatient Department of Government Ayurvedic Hospital, Ahmedabad, India. The protocol for carrying out the clinical study was approved by the Director, Department of Ayurveda and Homeopathic Medicine, Government of Gujarat, India and also by the institutional ethics committee for the clinical study. Informed consent was obtained from all patients enrolled in the study.

Patients having symptoms of bronchial asthma such as dyspnoea, wheezing, tightness in chest, and cough were enrolled in the study. Patients having breathlessness due to cardiovascular disorders, having very severe bronchial asthma (peak expiratory flow rate [PEFR] <20%, forced expiratory volume in one second [FEV1] <20% of predicted value) or having pulmonary tuberculosis (confirmed by chest screening), cardiovascular disorders, and pregnant women were excluded from the study.

The patients satisfying the inclusion and exclusion criteria were examined for baseline demographic data. Respiratory functions were recorded with the help of spirometer and blood samples were withdrawn for hematological examination before and subsequently at the end of 3 weeks treatment with *M. oleifera*. Patient was asked to keep mouthpiece of spirometer in mouth and hold it firmly with the help of lips. Patient was asked to breathe normally a few times then to take in a deep inspiration, as much as he/she can. Then, he/she is immediately instructed to blow out as hard and as fast as possible and keep breathing out till he can do so no more. The spirogram and the values of lung volumes and lung flow rates obtained were recorded. The whole process was repeated three times and the best results were recorded. Parameters assessed were forced vital capacity (FVC), forced expiratory volume in one second (FEV1), peak expiratory flow rate (PEFR), and forced expiratory flow rate between 25 and 75% (FEF_25-75%_). Maximum ventilatory volume (MVV) was determined by asking the patient to breathe as hard and fast as possible for 1 min.

The patients were given fine powder of dried seed kernels in a dose of 3 g bid for 3 weeks and were advised to take it with water. Patients were given medication supply for 1 week and were asked to report every week. At weekly visit, patients were asked for occurrence of any untoward effect (if any) and improvement in the symptoms observed. Symptom score was measured for all commonly observed symptoms of bronchial asthma before starting the treatment and at the end of 3 weeks of treatment. Score was graded as 3, 2, 1, and 0 for the presence of severe, moderate, mild, and the absence of any symptom, respectively.[9]

### Statistical analysis

The values were expressed as mean ± SEM. Data were analyzed using Student's paired t-test. The value of ‘*P*’ less than 5% (*P* < 0.05) was considered to be significant.

## Results

**Figure 1:**
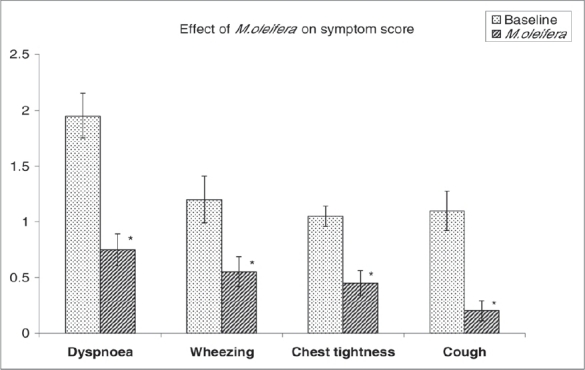
Effect of *M. oleifera* on symptom score. Each bar represents mean ± SEM. *Significantly different from baseline values (*P* < 0.05) (Student's paired t-test)

Baseline characteristics like age, height, weight, pulse rate, blood pressure, etc. of patients are shown in Table 1. Habit of smoking was found to be prevalent to the extent of 86% among asthmatic male patients. Also 25% of patients were found to have family history of asthma. Twenty percent of patients were found to have extrinsic (allergic) asthma and 80% were found to have intrinsic (nonallergic) type of asthma. There was significant decrease in symptom score of all the commonly observed symptoms of bronchial asthma [Figure 1]. The hematological parameters were not changed markedly by treatment with *M. oleifera.* However, the majority of patients showed significant increase in Hb values and ESR was significantly reduced [Table 2].

**Table 1:** Baseline characteristics of patients enrolled for the pulmonary function test (PFT) of ***Moringa oleifera***

*Variables*	*Mean ± SEM*	*Range*
Age (years)	33.4 ± 3.48	17-70
Male/Female	14/06	-
Height (inches)	63.64 ± 0.66	58-70
Weight (kg)	51.96 ± 1.32	38-64
Heart rate (beats/min)	75.72 ± 1.77	56-90
Asthma, duration (year)	5.26 ± 1.35	1-20
Systolic BP (mm Hg)	110.2 ± 2.58	95-150
Diastolic BP (mm Hg)	74.2 ± 2.56	60-110

**Table 2:** Effect of ***Moringa oleifera*** on hematological profile of patients

*Variables*	*Baseline values*		*After treatment*	
		
	*Mean ± SEM*	*Range*	*Mean ± SEM*	*Range*
Hb (g %)	11.54 ± 0.18	10-13.5	11.90 ± 0.18*	10-13.5
TC (per c mm)	7584 ± 226.54	5400-10400	6968 ± 196.28	5400-9800
Neutrophil (%)	71.44 ± 5.0	60-81	67.8 ± 1.0	60-77
Lym. (%)	26.68 ± 1.07	16-38	23.04 ± 1.00	12-34
Eosi. (%)	1.8 0 ± 0.14	1-3	1.28 ± 0.09	0-2
Mono. (%)	1.44 ± 0.14	0-2	1.24 ± 0.08	0-2
ESR (mm/h)	17.4 ± 2.28	5 - 40	11.00 ± 1.47*	5-35

* Significantly different from baseline (*P* < 0.05) (Student's paired t-test)

Spirometry tests before and after 3 weeks of treatment with *M. oleifera* revealed significant increase in FVC and FEV1. Mean % increases in FVC and FEV1 were 32.97 ± 6.03% and 30.05 ± 8.12%, respectively [Table 3]. Improvement was also observed in % predicted values. Twenty-five percent of patients were having predicted values of FVC above 80% before treatment with *M. oleifera*. After treatment with *M. oleifera*, 40% of patients were found to have predicted values of FVC above 80% [Table 4]. Out of 20 patients, 50% of patients were having FEV1 <60% of predicted values before treatment with *M. oleifera*, which was decreased to 35% of patients [Table 4]. Significant improvement in PEFR, FEF_25-75%_, and MVV was observed with *M. oleifera*. Mean % increase in PEFR was 32.09 [Table 3]. Forty percent had PEFR in the range of 20-40% of the predicted values before treatment with *M. oleifera*. After treatment, this was decreased to 20% of patients having PEFR <40% of predicted values [Table 4].

**Table 3:** Effect of ***Moringa oleifera*** on lung functions

*Parameters*	*Before treatment (Mean ± SEM)*	*After treatment (Mean ± SEM)*	*% Increase (Mean ± SEM)*
FVC (l)	1.942 ± 0.241	2.365 ± 0.220***	32.97 ± 6.03
FEV1 (l)	1.592 ± 0.251	1.896 ± 0.246**	30.05 ± 8.12
FEV1/FVC (%)	70.546 ± 6.083	73.656 ± 5.531	6.112 ± 2.555
PEFR (l/s)	3.982 ± 0.541	4.758 ± 0.516***	32.09 ± 11.75
FEF_25-75%_ (l/s)	2.143 ± 0.57	2.22 ± 0.489	20.04 ± 10.64
MVV (l/min)	34.04 ± 2.76	46.97 ± 5.49**	34.95 ± 8.44

** *P* < 0.01,

*** *P* < 0.001 (Student's paired t-test)

**Table 4:** Effect of ***Moringa oleifera*** on % predicted values

*% Predicted values*	*Forced vital capacity (% of patients)*		*Forced expiratory volume in one second (% of patients)*		*Peak expiratory flow rate (% of patients)*	
			
	*Baseline*	*M. oleifera*	*Baseline*	*M. oleifera*	*Baseline*	*M. oleifera*
20-40	10	10	15	10	40	20
40-60	35	20	35	25	25	30
60-80	30	30	20	25	20	10
80-100	25	40	30	40	15	40

## Discussion

The results from our earlier preliminary clinical study on *M. oleifera* suggest that there was appreciable decrease in severity of symptoms of asthma and also simultaneous improvement in lung function parameters.[10] The results of the effect of *M. oleifera* on four basic symptoms of bronchial asthma (dyspnoea, wheezing, chest tightness, and cough) revealed that score of all symptoms was reduced significantly. According to Unani medical theory, obstructive breathing may be due to a phlegmatic (thick sticky sputum) condition and it is produced mainly in those patients who have phlegmatic temperament. *Moringa oleifera* fruit is reported to cure *kapha*.[11] Our results support the effectiveness of *M. oleifera* in ameliorating the symptoms of bronchial asthma. The results of hematological parameters revealed that the majority of patients showed significant increase in their Hb values. These observations support earlier report that *M. oleifera* increases Hb levels in rats.[12]

Apart from the improvement in symptoms, *M. oleifera* was found to significantly improve FVC and FEV1. Forced vital capacity is clinically useful as an index of lung function. Forced expired volume in one second is a useful measure of how fast one can breathe out and full lungs can be emptied. It is the best measure of lung function for assessing airflow limitation or asthma severity.[13] *Moringa oleifera* also significantly improves PEFR, FEF_25-75%_, and MVV. Peak expiratory flow rate is a simple index of pulmonary function used in both research and clinical practice. Response to asthma treatment is usually accompanied by an increase in PEFR value and a decrease in its variability.[14]

Statistically significant increase in lung volumes (FVC and FEV1) and lung flow rates (PEFR, FEF_25-75%_, and MVV) suggest the usefulness of *M. oleifera* in the treatment of bronchial asthma.

Thus, the results of our study suggest that there was appreciable decrease in severity of symptoms of asthma and also simultaneous improvement in respiratory functions. No change in any general parameters and absence of any untoward effect during the course of study suggest the safety of drug in the dose used. Further, the hematological profile showed enhancement in the Hb level. Considering the easy availability, convenience, and its efficacy on oral administration, the seeds of *M. oleifera* may be considered as a useful drug for bronchial asthma. However, the detailed clinical and experimental studies are required to investigate its mechanism of action and therapeutic utility of these herbs.
